# Editorial: Understanding perinatal mental health psychiatric impact

**DOI:** 10.3389/fpsyt.2023.1118492

**Published:** 2023-02-07

**Authors:** Robert J. Wickham, Maria G. Van Pampus

**Affiliations:** ^1^Department of Psychology, Lafayette College, Easton, PA, United States; ^2^Department of Obstetrics and Gynecology, Onze Lieve Vrouwe Gasthuis, Amsterdam, Netherlands

**Keywords:** peripartum, depression, mental health, social support, perinatal development, psychiatry

## Introduction

Pregnancy and childbirth are typically viewed as positive life events, yet there are tremendous physical and psychological tolls undertaken by the pregnant mother. For example, the baseline rate of post-traumatic stress disorder (PTSD) following childbirth is estimated to be about 3% of the population, with up to 20% of mothers who are in an at-risk group developing PTSD (1, 2). Moreover, between 10 and 20% of pregnant women experience a mental health disorder, with depression being the most common (3). In general, there is a lack of diagnostical effort and treatments available for women experiencing mental health disorders during the perinatal period. The consequences of this lack of care are profound and include reductions in self-care, self-esteem, and general wellbeing, as well as a decreased capacity to bond or care for the newborn. To further explore the factors that contribute to mental health disorders during the perinatal epoch as well as the potential consequences of a lack of adequate mental health care, we organized this Research Topic of *Frontiers in Psychiatry* consisting of two themes.

## Theme 1: Factors contributing to maternal perinatal mental health disorders

Tamiru et al. explored the prevalence and factors of common mental health disorders in a very understudied part of the world: rural eastern Ethiopia. In this part of the world, they found that 37.5% of all pregnant women had a mental health disorder of some kind. Importantly, current substance abuse, nulliparity, gestational age (with mental health disorders occurring more likely in the first and third trimester relative to the second trimester), history of abortion, and lack of antenatal care were all significantly associated with mental health disorders during pregnancy. This study highlights the importance of regular screening programs for maternal mental health conditions, especially in low-income, rural communities, and recommends integrating these screenings into routine primary care visits to reduce the high relative risk in this population. Nakamura et al. showed that mothers who had high levels of harm avoidance—a personality trait typically characterized as excessive worrying, shyness, high levels of pessimism, and being highly fearful (4)—were more likely to develop post-partum depression. Importantly, this study showed that low social support was an important mediating factor between harm avoidance and post-partum depression. Mothers with high levels of self-directedness—defined as the ability of an individual to adapt, regulate, and control behavior to align with their chosen goals and values (5)—had a lower likelihood of post-partum depression and this was not mediated by levels of social support. These data suggest that a certain slice of the population that expressed higher harm avoidance would benefit from extra social support, which could be first identified through better screening by obstetricians and second supported through either family awareness of high harm avoidance in the mother or through social support groups. However, it should be noted that identifying personality traits generally is quite time and resource intensive. Relatedly, Schobinger et al. conducted interviews with newly post-partum mothers and their male partners to get a sense of the kind of support needed for new parents. They identified eight themes specific to post-partum mothers, such as concerns about: (1) “experiencing post-partum changes,” (2) “emotional needs,” (3) “creating a family unit,” (4) “self-esteem,” (5) “difficulty in communicating their needs,” (6) “post-partum stay,” (7) “returning home,” and (8) “to care for their newborn.” Given the diversity of concerns by post-partum women, it is likely that various forms of social support are required to ensure these concerns are adequately addressed.

## Theme 2: Potential biological effects of maternal mental health disorders and lack of social support network on child development

When parents are struggling with mental health disorders or extreme stressors, children sometimes bear some of this burden. Maternal deprivation, in this context defined as reduced care by the mother to her child, is one of the possible outcomes for mothers struggling with mental health disorders or extreme stress. Guo et al. demonstrated that when newborn rats were deprived of their mothers, they showed comparable levels of anxiety and stress in adulthood as rats who underwent chronic, unpredictable stress during adolescence. Moreover, pups that were maternally deprived or who later underwent chronic, unpredictable stress showed similar increases in methylation of the DRD2 promotor within the ventral tegmental area, a critical neural hub implicated in anxiety and depression (6, 7). These data suggest that maternal deprivation can produce comparable neurobiological changes and subsequent neuropsychiatric disorders, such as anxiety and depression. Greater social support for post-partum mothers would help to reduce the likelihood of maternal deprivation from occurring, especially in mothers who are more susceptible to mental health disorders.

It is well-known that extreme stress can weaken immune responses and increase susceptibility to illness. A lack of adequate social support for the pregnant mother could contribute to a weakened immune system due to increased stress. When pregnant mothers get ill, maternal immune activation (MIA) can sometimes have effects on the developing fetus, with higher incidences of autism spectrum disorder and schizophrenia (8). This is thought to be due to cytokines crossing the placental barrier and entering the developing fetal brain. Usui et al. showed that in a maternal immune activation (MIA) mouse model, a novel silicon-based hydrogen-producing antioxidant reduced the expression of inflammation-associated genes *Ifna1* and *Il-6* in the developing mouse brain and rescued the social communication (vocalization) deficits in neonatal mice produced by MIA. While this potential therapy is promising, this work underscores the importance of reducing severe illness during pregnancy, which can be mitigated by a stronger social support network.

## Conclusion

Mental health screening of mothers has its own challenges, such as feasibility, tool validity, and resources to provide appropriate care (9). However, a lack of maternal mental health care, including social support, can have devastating outcomes for both the family and the newborn. The work in this collection points toward a call to improve peripartum mental health care as a means to improve both the mental health of peripartum mothers as well as the incoming newborn. There is a great disparity in mental health disorders among more rural, low-income communities worldwide, and integrating, at a minimum, inquiry into the mother's mental health and wellbeing as part of the routine obstetric evaluation would be one step toward reducing this outcome gap.

## Author contributions

All authors listed have made a substantial, direct, and intellectual contribution to the work and approved it for publication.

## References

[1] Ayers S Bond R Bertullies S Wijma K. The aetiology of post-traumatic stress following childbirth: a meta-analysis and theoretical framework. Psychol Med. (2016) 46:1121–34. 10.1017/S0033291715002706 26878223

[2] Yildiz PD Ayers S Phillips L. The prevalence of posttraumatic stress disorder in pregnancy and after birth: a systematic review and meta-analysis. J Affect Disord. (2017) 208:634–45. 10.1016/j.jad.2016.10.009 27865585

[3] Sufredini F Catling C Zugai J Chang S. The effects of social support on depression and anxiety in the perinatal period: a mixed-methods systematic review. J Affect Disord. (2022) 319:119–41. 10.1016/j.jad.2022.09.005 36108877

[4] Chen CY Lin SH Li P Huang WL Lin HY. The role of the harm avoidance personality in depression and anxiety during the medical internship. Medicine. (2015) 94:e389. 10.1097/MD.0000000000000389 25590843 PMC4602540

[5] Smith DJ Duffy L Stewart ME Muir WJ Blackwood HD. High harm avoidance and low self-directedness in euthymic young adults with recurrent, early-onset depression. J Affect Disord. (2005) 87:83–9. 10.1016/j.jad.2005.03.014 15967233

[6] Addy NA Nunes EJ Wickham JR. Ventral tegmental area cholinergic mechanisms mediate behavioral responses in the forced swim test. Behav Brain Res. (2015) 288:54–62. 10.1016/j.bbr.2015.04.002 25865152 PMC4443858

[7] Small KM Nunes E Hughley S Addy AN. Ventral tegmental area muscarinic receptors modulate depression and anxiety-related behaviors in rats. Neurosci Lett. (2016) 616:80–5. 10.1016/j.neulet.2016.01.057 26828299 PMC4798862

[8] Estes ML McAllister AK. Maternal immune activation: implications for neuropsychiatric disorders. Science. (2016) 353:772–7. 10.1126/science.aag3194 27540164 PMC5650490

[9] The Lancet. Screening for perinatal depression: a missed opportunity. Lancet. (2016) 387:505. 10.1016/S0140-6736(16)00265-8 26867429

